# A Surface-Enhanced Raman Spectral Library of Important Drugs Associated With Point-of-Care and Field Applications

**DOI:** 10.3389/fchem.2019.00706

**Published:** 2019-10-25

**Authors:** Stuart Farquharson, Carl Brouillette, Wayne Smith, Chetan Shende

**Affiliations:** Real-Time Analyzers, Inc., Middletown, CT, United States

**Keywords:** opioids, buprenorphine, cannabis, fentanyl, SERS, drug analysis, trace analysis, spectral library

## Abstract

During the past decade, the ability of surface-enhanced Raman spectroscopy (SERS) to measure extremely low concentrations, such as mg/L and below, and the availability of hand-held Raman spectrometers, has led to a significant growth in the number and variety of applications of SERS to real-world problems. Most of these applications involve the measurement of drugs, such as quantifying medication in patients, identifying illicit drugs in impaired drivers, and more recently, identifying drugs used as weapons. Similar to Raman spectroscopy, most of the point-of-care and field applications involve the identification of the drug to determine the course of action. However, unlike Raman spectroscopy, spectral libraries are not readily available to perform the necessary identification. In a large part, this is due to the uniqueness of the commercially available SERS substrates, each of which can produce different spectra for the same drug. In an effort to overcome this limitation, we have measured numerous drugs using the most common, and readily available SERS material and hand-held Raman analyzers, specifically gold colloids and analyzers using 785 nm laser excitation. Here we present the spectra of some 39 drugs of current interest, such as buprenorphine, delta-9 tetrahydrocannabinol, and fentanyl, which we hope will aid in the development of current and future SERS drug analysis applications.

## Introduction

One of the most important applications of analytical chemistry is the analysis of drugs. Raman spectroscopy became an important tool for this application in its ability to quantify active and inactive pharmaceutical ingredients in manufactured products beginning in the 1990s (Tensmeyer and Heathman, 1989; Tudor et al., 1990; Cutmore and Skett, 1993; Petty et al., 1996; McCreery et al., 1998; Fini, 2004), and its ability to identify illicit and counterfeit products sold over the internet in the past two decades (Ryder et al., 1999; Carter et al., 2000; Bell et al., 2004; de Veij et al., 2008; Sacré et al., 2010; Lanzarotta et al., 2017). However, three significant events have occurred in the past 5 years that require the analysis of trace amounts of drugs. First, the over prescription of opioids contributed to 63,000 overdose fatalities in 2016 (Media Relations, 2018). Second, fentanyl is illegally entering the USA (U.S. Customs and Border Protection, 2019), where it is added to cocaine and heroin, contributing to approximately one third of the 2016 drug-related fatalities (Jones et al., 2018), and as of 2019 it is considered a form of terrorism by the USA Department of Homeland Security (DHS) (Hoffman et al., 2019). Third, the legalization of marijuana for medical, as well as recreational use in many states (Sanders, 2018) and cannabidiol for purported health benefits (Kelman and Sharp, 2018), has led to their ever increasing legal and illegal use in everyday products, such as food, beverages, and oils (Sanders, 2019). In the first case, ambulance and hospital physicians need methods to rapidly identify and quantify opioids in overdose patients. In the second case the DHS needs simple and fast methods to detect fentanyl in merchandise entering the country. In the third case the Food and Drug Administration needs methods to verify the identity and quantity of cannabinoids in medical, recreational, as well as new food and beverage products. The ability of surface-enhanced Raman spectroscopy (SERS) to measure extremely low concentrations, such as mg/L and below, could soon be the method of choice for these trace drug analysis needs.

The potential of SERS to perform such analyses began with the measurement of nitrogen- and sulfur-containing drugs in the late 1980s and early 1990s (Torres and Winefordner, 1987; Sutherland et al., 1990). The first forensic samples were measured at the turn of the century (Perez et al., 1998; Angel et al., 1999; Sägmüller et al., 2001; Faulds et al., 2002; Pînzaru et al., 2004; Ryder, 2005), followed quickly by measurement of drugs in body fluids (Perez et al., 1998; Farquharson and Lee, 2000; Trachta et al., 2004a). We began measuring chemotherapy and illicit drugs in saliva a few years later (Farquharson et al., 2005, 2011; Shende et al., 2005; Inscore et al., 2011; Dana et al., 2015), and more recently, measuring opioid treatment drugs in the saliva of USA military veterans (Farquharson et al., 2017, 2019), as well as fentanyl in saliva and blood (Shende et al., 2019).

During the development of these applications we noticed a lack of SERS publications for these and other drugs of interest. Furthermore, most of the available spectra were obtained using author-unique substrates and various excitation wavelengths that resulted in spectra that are incomparable. Therefore, we measured 39 drugs of interest using the most common and readily available SERS-active material, gold colloids, and using the most common excitation wavelength of portable Raman spectrometers, 785 nm. Here we present a spectral library of numerous opioids, illicit and treatment drugs, as well as some important metabolites, which would be suitable for identification of samples using either field or point-of-care Raman spectrometers.

## Materials and Methods

All solvents and chemicals used to prepare samples and colloids were obtained from Sigma-Aldrich (St. Louis, MO). The drugs used to prepare the spectral library were purchased as 1 mg/mL acetonitrile or methanol certified forensic samples obtained from Cerilliant Corp (Round Rock, TX). Drug samples were prepared by diluting the forensic samples by a factor of 5 in HPLC grade water and then added to an equal volume of colloid to make 0.1 mg/mL (100 ppm) initial concentrations, which were in some cases further diluted with water (see figure captions). The gold colloids used for SERS were synthesized following a modified Lee-Meisel method (Lee and Meisel, 1982). Briefly, a solution of 0.005 M HAuCl_4_ (100 mL), was slowly added to 300 mL of 0.02 M NaBH_4_ in an ice bath. A solution of 1% polyvinyl alcohol (50 mL) was added and refluxed for 1 h. 0.5 M NaCl was used as an aggregating agent. The solutions were placed in 2 mL glass vials from Glass Vials Company (Hanover, MD) and then measured using ~30 mW of 785 nm laser excitation with an in-house Raman spectrometer and collection software (RTA LabRaman and Vista). Each presented spectrum is the average of five 1-s integrations. In some cases a glass sample vial spectrum was subtracted to flatten the baseline between 1,100 and 1,700 cm^−1^. All spectral peaks are given to the nearest 5 cm^−1^.

## Results and Discussion

The spectra are, in general, presented in the following order: opioids, synthetic opioids, stimulants, sedatives, cannabinoids, and common drugs. A basic background is provided for each drug to provide a framework for why its analysis at low concentrations is important. Each figure, as best possible, contains three drugs that share the same basic chemical structure so that their spectra can be compared.

### Opioids

In simplest terms opioids influence the release and uptake of neurotransmitters at one or more of the delta-, mu-, kappa-, and zeta-opioid receptors (Corbett et al., 2006), involved in the reward (pleasure) pathway in the brain and the pain pathway in both the brain and spinal cord. The opioids have structures similar to the neurotransmitter dopamine associated with the reward pathway and the neuropeptide endorphins associated with the pain pathway, allowing them to bind to sites on the end of neurons. The effectiveness of the various opioids is also a function of their lipophilicity, the ability to pass through the blood-brain barrier, and their reactivity with the neuron sites associated with the release and uptake of these and other neurotransmitters and neuropeptides. These abilities make opioids ideal for treating depression and pain. Unfortunately, the activity level of these neural chemical and biochemical interactions diminish with repeated opioid use, such that greater amounts are needed to achieve previous levels of pleasure and/or pain relief, which can lead to addiction and dependence.

While the pain relieving powers of opium have been known for thousands of years, the primary active drugs, morphine and codeine, were not isolated until the early 1800s (Courtwright, 2009; Newton, 2015). Today these two natural drugs are used to produce a wide range of semi-synthetic opioid medicines, such as hydrocodone, hydromorphone, oxycodone, and oxymorphone, treatment medicines, such as buprenorphine and naloxone, and the illicit drug heroin. More recently, much more powerful opioids have been synthesized, such as fentanyl and carfentanil.

Heroin is a Schedule I drug, as it does not have any accepted medical use and it has a high potential for abuse (Brandán, 2018). Heroin use resulted in nearly 16,000 deaths in the USA in 2017 (NIH, 2019). It is synthesized by acetylation of morphine, and the product usually contains some 6-acetylcodeine, due to the presence of codeine in the starting solution, as an impurity (O'Neal et al., 2001). 6-acetylcodeine measured in urine is often used to distinguish between heroin use and prescription opioid use (Staub et al., 2001).

**Figure 1:**
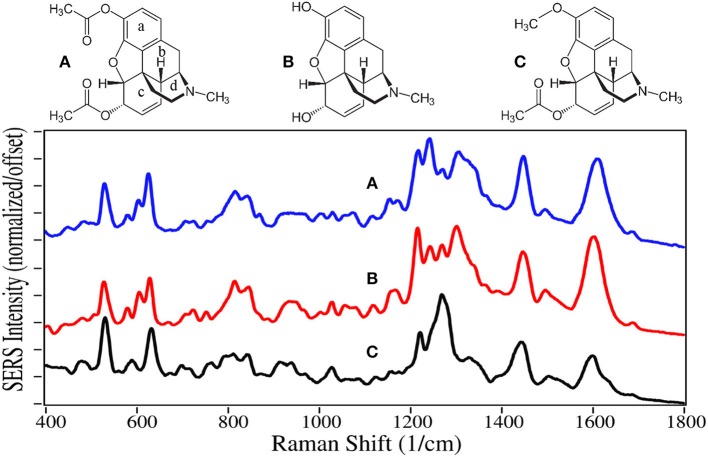
Structures and 10 ppm SERS of **(A)** heroin, **(B)** morphine, and **(C)** 6-acetylcodeine.

The SERS of heroin, morphine, and 6-acetylecodeine are dominated by peaks at 530, 625–630, 1,215–1,225, 1,270–1,275, 1,440–1,445, and 1,600–1,610 cm^−1^ (Figure 1). Based on their similar Raman spectra (Rana et al., 2010; Gardner et al., 2013), these peaks are assigned to c-ring out-of-plane bending plus a- and c-ring CH bending, a-ring C = CH out-of-plane bending, c-ring CCC out-of-plane bending, c-ring CC stretching, d-ring CH_2_ scissoring, and c-ring CC stretching. These drugs can be differentiated primarily by their peak intensities between 1,200 and 1,400 cm^−1^. It is worth noting that the SERS of morphine presented here on gold has many of the same peaks as that reported for silver (Rana et al., 2010). However, the relative intensities are substantially different, likely due to the fact that morphine is protonated at pH 7 at very low concentrations in water [pKa_1_ is 8.21 (Lide, 1997)] and attracted more to electronegative gold than electropositive silver, affecting the orientation on the metal surface and interaction with the plasmon field. Furthermore, these orientation-induced spectral differences would challenge the ability of library search-and-match software.

**Figure 2:**
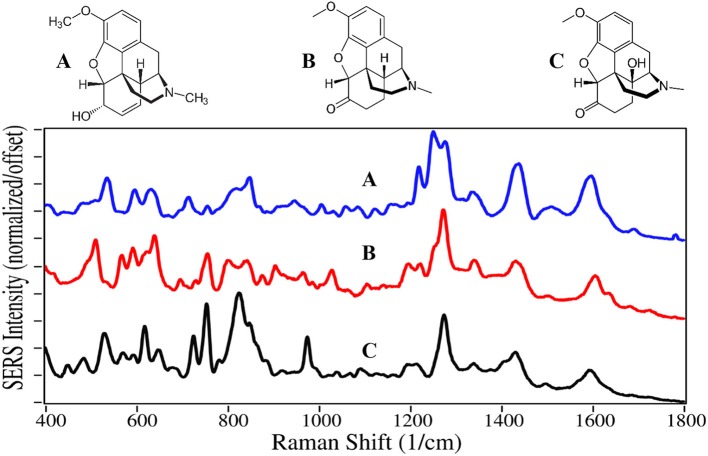
Structures and 100 ppm SERS of **(A)** codeine, **(B)** hydrocodone, and **(C)** oxycodone.

Codeine, hydrocodone, and oxycodone are Schedule II drugs usually prescribed in combination with nonsteroidal anti-inflammatory drugs, such as acetaminophen, aspirin, and ibuprofen, to treat varying degrees of pain. Oxycodone, sold as OxyContin, is currently the most abused opioid, and resulted in ~6,000 deaths in 2017 (Hedegaard et al., 2018; Pergolizzi et al., 2018). The SERS of codeine, hydrocodone, and oxycodone are also dominated by similar peaks at 510–530, 620–640, 1,275, 1,435, and 1,595–1,605 cm^−1^, and assigned to the same vibrational modes (Figure 2) (Rana et al., 2010). Substitution at the juncture of the c, b, and d rings influences the intensity of the CC and CCC bending modes. The drugs also have unique spectral features in the low frequency region between 500 and 850 cm^−1^, such as the c-ring CH out-of-plane bending mode at 535, 510, and 530 cm^−1^ for the three drugs, respectively. Again, spectral differences were obtained on silver (Rana et al., 2010).

Hydromorphone and oxymorphone are two additional Schedule II opioids used to treat pain, and often prescribed to alleviate pain due to cancer (Sloan, 2008; Pigni et al., 2011). The latter drug has proven highly addictive, and was removed from the market in 2017 at the request of the Food and Drug Administration (Peddicord, 2017).

**Figure 3:**
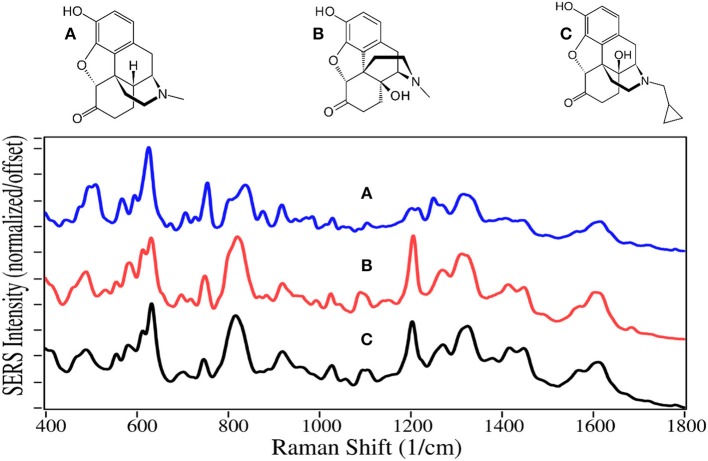
Structures and 100 ppm SERS of **(A)** hydromorphone, **(B)** oxymorphone, and **(C)** naltrexone.

**Figure 4:**
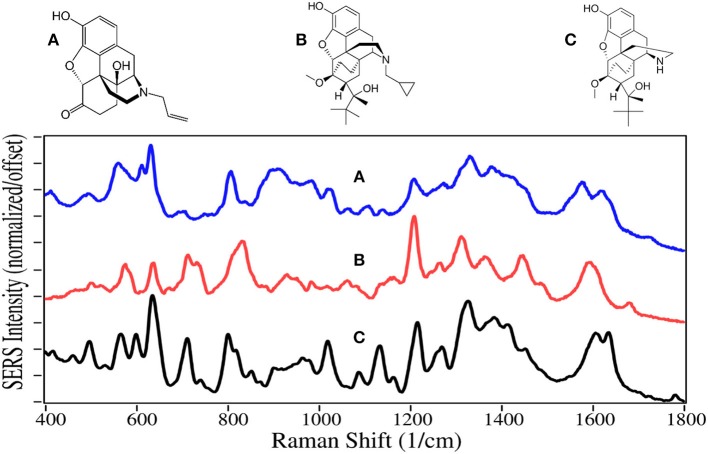
Structures and 100 ppm SERS of **(A)** naloxone, **(B)** buprenorphine, and **(C)** norbuprenorphine.

Not all opioids are used to treat pain and depression, some are used to treat opioid addiction. For example, the chemical structures of naltrexone, naloxone and buprenorphine, are very similar to the above described drugs, and can compete for or block the opioid receptor sites (Figures 3, 4). Naltrexone also has a much greater affinity for opioid sites than morphine, while not generating the euphoria of addicting opioids (Melichar et al., 2003), and is used to treat opioid addiction and overdose patients (Comer et al., 2006; Lynn and Galinkin, 2018). Hydromorphone, oxymorphone, and naltrexone, like codeine and morphine, contain SERS peaks at 625–635, 1,205–1,210, 1,270, 1,445–1,450, and 1,610–1,615 cm^−1^, and can be assigned to the same vibrational modes. They also have peaks at 485–490, 750, a single or double at 820, 1,030, a shoulder at 1,570, and a weak peak at 1,675–1,695 cm^−1^, assigned to a-/c-CH bending, out-of-plane C = O bending (Rana et al., 2010), c-ring CH bending, CH_2_ rocking, e-ring CN stretch (Socrates, 2001), and a c-ring C = C stretch (Rana et al., 2010). It is worth noting that the spectra of oxymorphone and naltrexone are nearly identical, suggesting that the methylcyclopropane functional group is either non-SERS-active, or directed away from the gold surface due to the attraction of the OH groups. The primary difference between the spectra of these two drugs is the relative peak intensities.

Naloxone is a prescription opioid treatment drug that has opioid site-blocking ability and minimal side-affects similar to naltrexone. However, its high lipophilicity allows it to rapidly pass through the blood-brain barrier, and when taken intravenously can arrest the effects of opioids in 2–5 min (Milne and Jhamandas, 1984). Consequently, it has become the drug of choice for emergency rooms and first responders to treat people who have overdosed, especially those taking fentanyl (Walsh, 2014). As of this year, a nasal spray has been approved for use (Meyer, 2019).

Buprenorphine, a Schedule III drug with a modest potential for abuse, is one of the most successful opioid treatment medications, approved for use in 1981 (Sadock et al., 2012). It has a substantially higher affinity to the mu-opioid receptor, involved in both the reward and pain pathways, but only partially activates the receptor, while it suppresses the kappa-opioid receptor and the associated psychological depression (Khroyan et al., 2015). It is 20–30 times as strong as morphine in relieving pain (DEA, 2013). Furthermore, its low lipophilicity allows it to stay in the cerebrospinal fluid, and coupled with its mean metabolic half-life of 36 h, it provides long-lasting effects that have made it the drug treatment of choice for substance use disorders (Arias and Kranzler, 2008; Urbano et al., 2014). For long term treatment of patients, buprenorphine is combined with naloxone, sold as Suboxone, to prevent the patient from transferring their addiction to buprenorphine (Yassen et al., 2007). One of the challenges in treating patients is compliance. Patients may give into withdrawal symptoms, and re-initiate opioid use. Recently, it has been shown that buprenorphine and its metabolite, norbuprenorphine, can be detected by SERS in saliva (Farquharson et al., 2017, 2019), while other studies indicate that their relative concentrations could be used to determine the time of dosing (Kronstrand et al., 2008).

Naloxone, buprenorphine, and norbuprenorphine, like morphine, contain SERS peaks at 630–640, 810–839, 1,205–1,220, and 1,595–1,620 cm^−1^, and can be assigned to the same vibrational modes. The spectra of these drugs are unique in the 475–900 cm^−1^ and 1,550–1,650 cm^−1^ regions. While the structures and hence SERS share a lot of similarity for all of these natural and semi-synthetic opioids, spectral search and match software should easily distinguish which one might be in a sample (Farquharson et al., 2011), especially if the search focuses on the 475 to 850 cm^−1^ and the 1,175–1,375 cm^−1^ regions. However, quantifying mixtures would be difficult at best.

Methadone and meperidine, Schedule II drugs, were two of the first fully synthetic opioids, developed in the 1930s to overcome shortages of codeine, morphine and their semi-synthetic products. While methadone interacts with opioid receptors to alleviate pain, it is primarily used today for opioid maintenance therapy, as it is slow acting with a mean elimination half-life of 22 h (Eap et al., 1999). Meperidine, also known as pethidine and sold as Demerol, was the drug of choice to treat pain during the middle of the last century. However, it was discovered that its metabolite, norpethidine, was toxic (Stone et al., 1993), and it has been replaced by safer drugs. Methylphenidate has a structure nearly identical to meperidine, but has a significantly different pharmacology. It blocks the reuptake of dopamine and norepinephrine by neuron receptors (Kimko et al., 1999), and consequently acts as a stimulant. It is sold as Ritalin to treat attention deficit hyperactivity disorder (ADHD), and has been highly prescribed in the USA during the first part of this century (Pharmaceutical Society, 2015).

**Figure 5:**
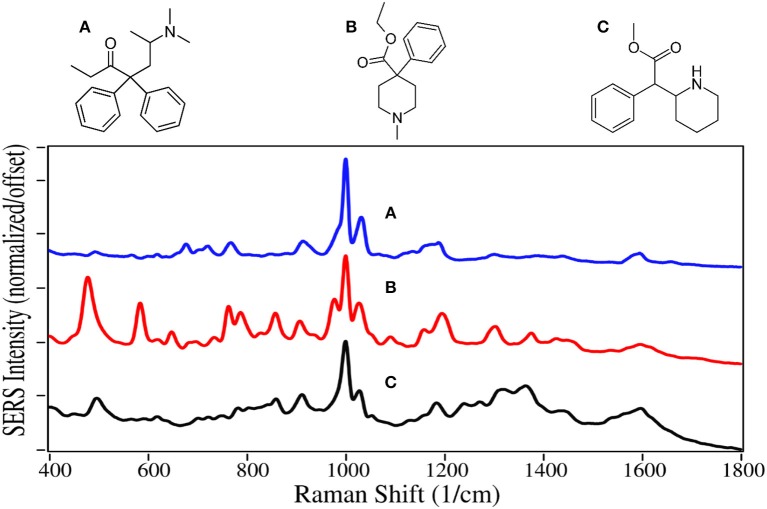
Structures and 100 ppm SERS of **(A)** methadone, **(B)** meperidine, and **(C)** methylphenidate.

Methadone, meperidine, and methylphenadate are all dominated by the symmetric and asymmetric phenyl ring breathing modes at 1,000 and 1,025–1,035 cm^−1^, and to some extent the trigonal phenyl ring breathing mode at 1,595–1,600 cm^−1^ (Figure 5) (Dollish et al., 1974; Carter et al., 2000). All three drugs have a peak at 910–915 cm^−1^ due to CNC stretching (Stanley, 2014). The same assignments have been given for SERS of methadone, except for the 1,660 cm^−1^ peak, which is assigned to a tertiary amine mode (Stanley, 2014), as opposed to a carbonyl mode that was very intense on silver (Trachta et al., 2004a). While meperidine and methylphenidate have nearly identical structures, the former appears to interact with the gold more strongly having more intense peaks, with many of the same peaks as the normal Raman spectrum (Angel et al., 1999).

In 1960, fentanyl, a Schedule II drug, was synthesized by Paul Janssen by modifying the structure of meperidine to make it more lipophilic so it would rapidly cross the blood-brain barrier, and consequently make its ability to ease pain more powerful (Stanley, 2014). He succeeded in making a drug ~100 times more potent than morphine. Today fentanyl is widely used as an anesthetic during surgery, which may include a benzodiazepine sedative, and naloxone after surgery to arrest fentanyl's effects (Comer et al., 2006). Unfortunately, it has become a recreational drug typically mixed with heroin, 250 pounds of which were seized at the Mexican border earlier this year (U.S. Customs and Border Protection, 2019). In 2016 it was responsible for ~20,000 overdose deaths in the USA (Jones et al., 2018). Furthermore, it and its analogs carfentanil and remifentanil, are considered weapons of war, as the latter two were used by the Russian military to incapacitate rebels in a Moscow theater (Wax et al., 2003; Riches et al., 2012). In 2016 Canadian authorities seized 1 kg of carfentanil sent from China, equivalent to 50 million lethal doses (Kinetz and Butler, 2016). Fortunately, the Chinese government has since banned its sale. While carfentanil's use is limited to tranquilizing animals, remifentanil is used in surgery similar to fentanyl.

**Figure 6:**
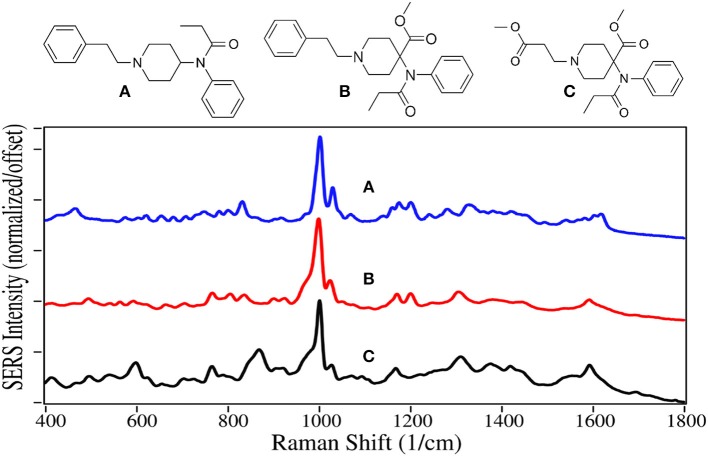
Structures and 10 ppm SERS of **(A)** fentanyl, **(B)** carfentanil, and **(C)** remifentanil.

SERS for all of these drugs are dominated by the symmetric and asymmetric phenyl ring breathing modes at 1,000 and 1,025–1,035 cm^−1^, and a modest trigonal phenyl ring breathing mode at 1,595–1,600 cm^−1^ (Figure 6) (Stanley, 2014; Leonard et al., 2017). All three drugs also have peaks at 590–620, 830 to 870, and 1,310–1,320 cm^−1^, which are assigned to a CCC phenyl bending mode (Hummel and Unterwald, 2002), a piperidine C-C mode or out-of-plane phenyl CH stretch, and a piperidine CH mode (Stanley, 2014). While the spectra are very similar, each has several unique features for identification.

### Stimulants

This class of drugs also acts upon the central nervous system, primarily by preventing the uptake of the dopamine, serotonin, and norepinephrine (adrenaline) neurotransmitters. This causes their accumulation in the synaptic gap, and thereby continuously stimulates the reward and cognitive (alertness) pathways (Hummel and Unterwald, 2002), and increases blood pressure and heart rate (Zimmerman, 2012). Consequently, these drugs can be highly addictive and dangerous.

Cocaine is a natural product isolated from the coca plant, and has chemical functional groups similar to natural and synthetic opioids, and as such affects the central nervous system as described above. It is the second most used illegal drug in the world behind cannabis (Karila et al., 2014). Approximately 15 million people in the USA use cocaine yearly (Pomara et al., 2012), which resulted in ~14,000 fatalities in 2017. The primary metabolite of cocaine is benzoylecgonine, which is used to test for cocaine use, as it is present in urine for as long as a week (Schindler and Goldberg, 2012). In contrast, cocaethylene is often tested for in autopsy, since it is produced when cocaine and alcohol are used together, a combination that increases the chance of death by “18 to 25 fold” compared to cocaine taken by itself (Andrews, 1997; Pennings et al., 2002).

**Figure 7:**
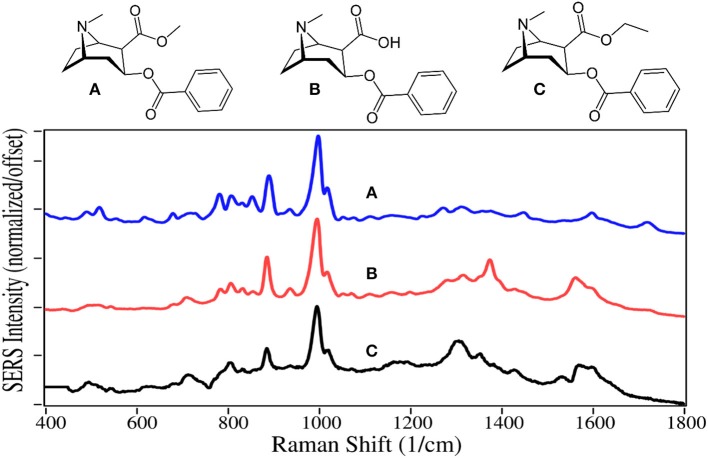
Structures and 100 ppm SERS of **(A)** cocaine, **(B)** benzoylecgonine, and **(C)** cocaethylene.

Cocaine, benzoylecgonine, and cocaethylene are also dominated by the symmetric and asymmetric phenyl ring breathing modes at 995–1,000 and 1,015–1,020 cm^−1^, and to some extent the trigonal phenyl ring breathing mode at 1,595–1,600 cm^−1^ (Figure 7). Another intense mode appears at 885–890 cm^−1^ due to the tropine ring stretch (Carter et al., 2000; D'Elia et al., 2016). The primary difference in their spectra occur in the 800 to 900 cm^−1^ region due to the changes in the ester group, which also appears to influence the intensity of the weaker tropine modes between 1,300 and 1,400 cm^−1^. It is also worth noting that the 1,600 cm^−1^ trigonal phenyl ring breathing mode and ester carbonyl stretch at 1,720 cm^−1^ are more intense when measured with silver (Dana et al., 2015).

Amphetamine and methamphetamine are Schedule II synthetic drugs, while mephedrone is a Schedule I synthetic drug. All three have chemical structures similar to dopamine, serotonin and epinephrine, and much like cocaine, cause accumulation of these neurotransmitters in the synaptic gap stimulating the reward and cognitive pathways (Arnold, 2000). A 25/75% mixture of the amphetamine enantiomers, prescribed as Adderall, is used to treat ADHD (Bidwell et al., 2011). Amphetamine is also used to enhance athletic performance. While methamphetamine can also be used for these applications with deminished effect, it is primarily used as a recreational aphrodisiac. Mephedrone, also known as “bath salts” and “meow meow” (Glennon, 2014), is used as a recreational drug with effects similar to the amphetamines and cocaine.

**Figure 8:**
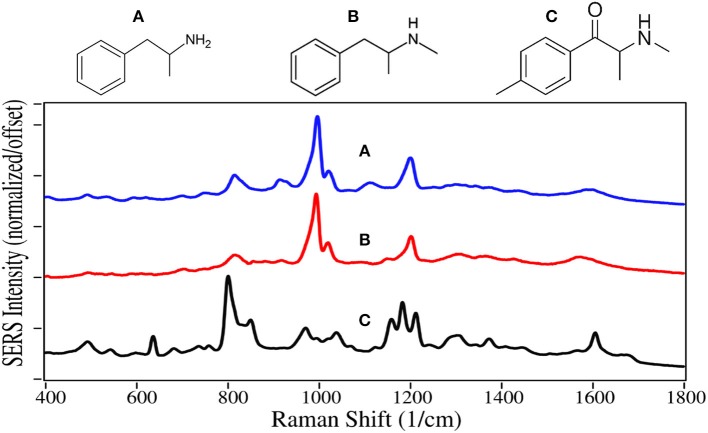
Structures and 100 ppm SERS of **(A)** amphetamine, **(B)** methamphetamine, and **(C)** mephedrone.

As with fentanyl and cocaine and their analogs, amphetamine and methamphetamine are dominated by the symmetric and asymmetric phenyl ring breathing modes at 995–1,000 and 1,020 cm^−1^, and to some extent the trigonal phenyl ring breathing mode at 1,595–1,600 cm^−1^ (Figure 8) (Carter et al., 2000; Hargreaves, 2013). The peaks at 815–820 and 1,200 cm^−1^ are C-ring modes for the amphetamines, which are enhanced as the para-substituted C-ring-C modes for mephedrone (Carter et al., 2000). In contrast, the para-substituted benzene ring of mephedrone results in intense peaks at 805 and 1,215 cm^−1^, and an asymmetric C-N-C stretch at 1,185 cm^−1^ and amide mode at 1,672 cm^−1^ (Milne and Jhamandas, 1984). Similar SERS has been reported for methamphetamine (Sägmüller et al., 2003) and mephedrone (Mabbott et al., 2013).

3,4-Methylenedioxyamphetamine (MDA) 3,4-Methylenedioxymethamphetamine (MDMA) and 3,4-Methylenedioxy-*N*-ethylamphetamine (MDEA) are all Schedule I drugs because they are used recreationally and have no medical value. All three drugs have structures similar to the amphetamines and dopamine, with the addition of a dioxole ring. They not only inhibit reuptake, but also enhance release of dopamine, serotonin and epinephrine, stimulating the reward and cognitive pathways. The result is enhanced euphoria and psychedelic effects for these drugs, also known as “the love drug,” “ecstasy,” and “Eve,” respectively.

**Figure 9:**
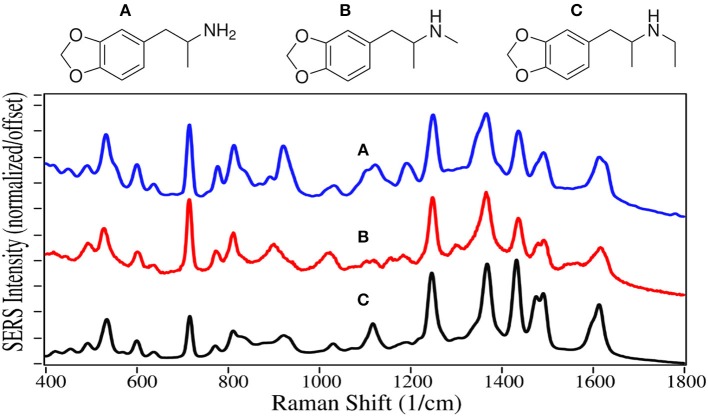
Structures and 100 ppm SERS of **(A)** MDA, **(B)** MDMA, and **(C)** MDEA.

The SERS of MDA, MDMA and MDEA are very similar to each other with 7 intense peaks occurring at 530–535, 715–720, 1,250, 1,365–1,370, 1,430–1,435, 1,470–1,480, and 1,620 cm^−1^, largely due to the dioxole ring alone or coupled with the phenyl ring (Figure 9). The first 3 peaks dominate the normal Raman and SER spectra on silver (Bell et al., 2000; Lombardi et al., 2013), but the remaining peaks, while present, are of much lower relative intensity.

### Sedatives

Most sedatives are benzodiazepines, which increase the effect of the neurotransmitter gamma-aminobutyric acid, which in turn increases the flow of chloride ions into the neuronal cell (Derry et al., 2004). This increases the neuron's chemical potential, such that it is less likely to fire, reducing both brain and spinal cord function (Date et al., 1984), making the user tired.

Diazepam, temazepam, and oxazepam are Schedule IV drugs primarily used to treat anxiety and sleeplessness. Diazepam easily crosses the blood-brain barrier, and has a long half-life of 30 to 56 h (Oelschläger, 1989). It was first marketed in 1963 as Valium, and rapidly became the highest prescribed drug in the USA (Calcaterra and Barrow, 2014). Temazepam is largley prescribed for people having trouble sleeping, while oxazepam is more for people who have trouble staying asleep. Both drugs are less powerful with shorter half-lives than diazepam. Since the reward pathway is not engaged by these drugs, they are not addicting, but dependence can occur, particularly when combined with alcohol (Poulos and Zack, 2004). In 2017 there were 11,500 deaths due to overdose, many involving automobile accidents (Smink et al., 2010).

**Figure 10:**
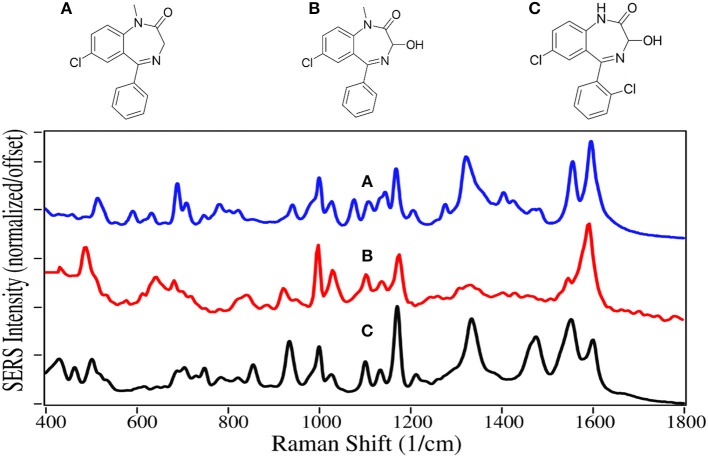
Structures and 100 ppm SERS of **(A)** diazepam, **(B)** temazepam, and **(C)** oxazepam.

The benzodiazepines are dominated by the 7-membered diazapine and phenyl ring modes. The former modes at 935–945, 1,170, and 1,590–1,600 cm^−1^ are due to ring stretching, C-H deformation, and ring stretching, respectively (Figure 10) (Neville et al., 1995). Diazepam and Oxazepam have phenyl modes at ~1,000, 1,030, 1,325–1,335, and 1,555 cm^−1^, whereas temazepam is missing the 1,330 and 1,555 cm^−1^ due to the chlorine substitution that reduces the symmetryof these vibrations. The SERS are similar to the normal Raman spectra of these drugs (Shende et al., 2014), as well as the SERS using silver (Cinta et al., 1999; Trachta et al., 2004b).

### Other Drugs

Delta-9 Tetrahydrocannabinol (THC), first isolated in 1964 (Pertwee, 2006), is the main psychoactive component of cannabis. Its C_22_ structure, similar to the C_22_ fatty acid neurotransmitter anandamide, allows it to bind and partially activate both the cannabinoid receptors located in the central nervous system and those in cells of the immune system. THC also indirectly influences the mu- and gamma-opioid receptors (Mechoulam and Fride, 1995). These actions result in modest euphoria, relaxation, and for some users, anxiety. Cannabidiol (CBD) represents 40% of the oil extracted from cannabis, making it economical for use in products (Hazekamp, 2018). However, it is not psychoactive (Iseger and Bossong, 2015), and the only verified medical use is the treatment of severe forms of epilepsy (FDA, 2018). Cannabinol (CBN) provides the same effects as THC, but to a lesser extent. It is also the primary metabolite of THC, and often tested in body fluids to determine use of either. Currently, 47 states allow medical use to varying degrees, and 10 states allow recreational use (Media Relations, 2018; State Medical Marijuana Laws, 2018).

**Figure 11:**
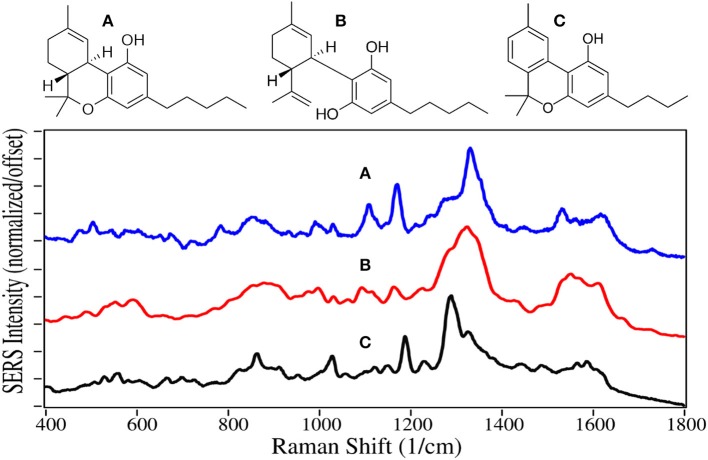
Structures and 10 ppm SERS of **(A)** tetrahydrocannabinol, **(B)** cannabidiol, and **(C)** cannabinol.

The phenyl ring peak intensities, despite being weak, appear for THC, CBD and CBN at 1,000, 1,030, and 1,610–1,615 cm^−1^, although the former two relative peak intensities for CBN are reversed (Figure 11). Peaks at 855 and 864 cm^−1^ for THC and CBN are assigned to stretching of the middle tetrahydropyran ring (Milne and Jhamandas, 1984; Stanley, 2014). Weak intensity peaks at 1,095–1,110 and 1,165–1,190 cm^−1^ are assigned to C-C stretching of the alkane chain for CBN, and C-C stretching of the rings, respectively, while a peak at 1,530–1,565 cm^−1^ is assigned to C = C stretching. The 1,250 to 1,350 cm^−1^ is the most interesting spectral region with two peaks at ~1,290 and 1,330 cm^−1^, both assigned to CH deformation modes. The latter peak appears to have significant contribution from the two CHs at the junction of the cyclohexene and tetrahydropyran rings. The same intensity difference has been observed for THC and CBN for their Raman spectra (Fedchak, 2014). While SERS of these drugs on gold are similar to Raman spectra, their SERS using silver are considerably different (Yüksel et al., 2016; Sivashanmugan et al., 2019).

Nicotine and caffeine are two of the oldest drugs, used as stimulants, and are largely unregulated. They are primarily produced by extraction from tobacco plants and coffee beans, respectively. Nicotine binds to acetylcholine receptors in the brain releasing several neurotransmitters, in particular dopamine (Malenka et al., 2009), and appears to also cause the release of natural opioids (Kishioka et al., 2014). It thus activates the reward pathway in the brain, making it highly addictive (Stolerman and Jarvis, 1995). The availability of traditional cigarettes and now e-cigarettes makes nicotine one of the most abused drugs (Sajja et al., 2016). Traditional cigarettes have been the dominant cause of lung cancer, and a major contributor to heart disease for the past 50 years (Nicotine, 2014). The intent of e-cigarettes, or vaping, was to reduce these horrendous side-effects. Unfortunately, vaping has grown rapidly among high school students to 21% in 2016 (Surgeon General, 2016), potentially leading to an even greater number of nicotine addicts.

Caffeine is legal in most of the world and is the most consumed psychoactive drug. Its mechanism is somewhat different than other stimulants, in that its molecular structure is similar to adenosine, and it binds to the adenosine receptors, but does not slow the release of neurotransmitters that regulate breathing, heart rate and blood pressure in the medulla oblongata (Fisone et al., 2004). Theophylline, which is also extracted from coffee beans, is structurally similar to caffeine, and it also binds to the adenosine receptors (Daly et al., 1987). It is mostly used to treat asthma and other respiratory conditions.

**Figure 12:**
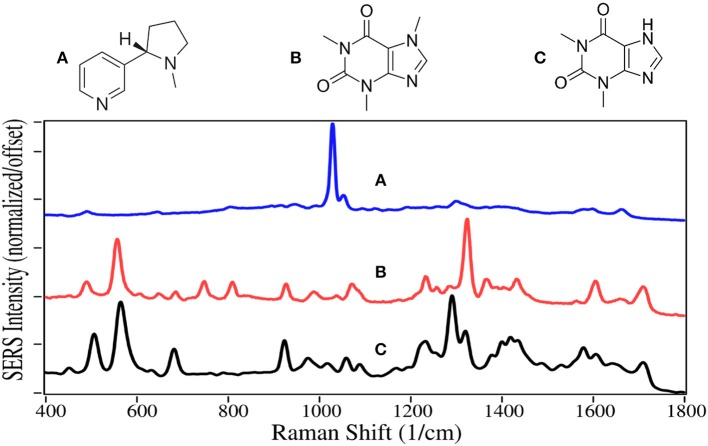
Structures and 100 ppm SERS of **(A)** nicotine, **(B)** caffeine, and **(C)** theophylline.

The SERS of nicotine is dominated by the symmetric pyridine ring breathing mode at 1,030 cm^−1^, which also produces peaks at 645, 1,055, and 1,595 cm^−1^, assigned to in-plane ring, trigonal ring breathing modes (Figure 12) (Milne and Jhamandas, 1984). These peaks are all the same in both Raman and SERS (Barber et al., 1994). However, the relative intensities more closely match Raman than SERS using silver (Itoh and Bell, 2017). The SERS of caffeine and theophylline are dominated by two peaks, one at 555–570 cm^−1^, the other at 1,290 and 1,320 cm^−1^, assigned to pyrimidine ring breathing and imidazole ring trigonal stretching, for theophylline and caffeine, respectively (Pavel et al., 2003). The shift in frequency is due to the methyl group on the imidazole ring for caffeine. The following peaks occur for both drugs, 490–510, 925, 1,235, 1,605, and 1,710 cm^−1^ assigned to an in-plane ring-ring deformation, deformation of both rings, HCN bending, C-C stretching, and in-phase C = O stretching. The SERS for theophylline is very similar to that reported for caffeine (Ricciotti and FitzGerald, 2011).

Acetaminophen, aspirin and ibuprofen inhibit cyclooxygenase from producing the prostaglandin hormones that are local messengers of pain, fever and inflammation (Day and Graham, 2004; Ricciotti and FitzGerald, 2011). However, acetaminophen only inhibits cyclooxygenase in the brain, and consequently, is not an anti-inflammatory. Aspirin also inhibits clotting by keeping blood platelets from sticking together. Each of the three drugs are often combined with other drugs, such as prescription opioids to aid in reducing pain. These three drugs are the most common medication used to treat pain in the world (WHO, 2015). Unfortunately, the high use of acetaminophen led to ~1,200 overdose deaths in the USA in 2016 (Hedegaard et al., 2018). Acetaminophen, aspirin, and ibuprofen were the 38th, 35th, and 17th most prescribed drugs in the USA in 2016, respectively (ClinCalc, 2019).

**Figure 13:**
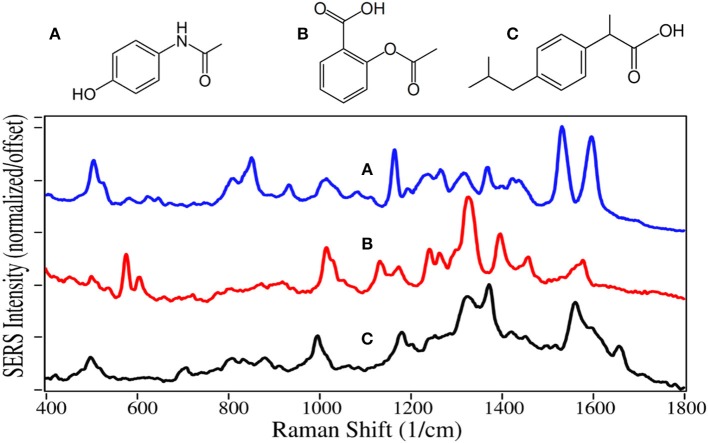
Structures and 100 ppm SERS of **(A)** acetaminophen, **(B)** aspirin, and **(C)** ibuprofen.

The SERS of acetaminophen has peaks at 810, 855, 1,235, 1,320, 1,535, and 1,610 cm^−1^, which are assigned to CNC ring stretching, ring breathing, C-C ring stretching, amide III, and amide II modes, respectively (Figure 13) (Diniz et al., 2004). Aspirin and ibuprofen have the typical phenyl peaks at 1,000–1,015, 1,325, and 1,580–1,595 cm^−1^. In addition to the 1,325 cm^−1^ peaks, they have intense peaks at 1,400 and 1,375 cm^−1^, respectively, possibly due to COO stretching (El-Shahawy, 1988; Vueba et al., 2008). Ibuprofen also has a carboxylic acid C = O peak at 1,660 cm^−1^, notably absent for aspirin. While the acetaminophen spectrum matches the Raman spectrum, the SERS of aspirin and ibuprofen are significantly different, especially their relative peak intensities. This is likely due to their different chemical interactions with gold and the resultant orientation.

The SERS peaks, according to wavenumber, for the 39 drugs are summarized in Table 1 in terms of vibrations common to each drug type, as well as 1 or 2 unique vibrations that could be used to identify and differentiate the drugs within each group. The peaks are arranged in columns such that vibrations can be compared across the entire set of 39 spectra. The references used to make the vibrational assignments are also included. However, for many drugs, the assignments were based on functional groups common to a referenced drug, as well as the measured peak wavenumber and intensity. It is worth noting that many spectral analysis algorithms can be used to both identify an unknown substance and quantify simple mixtures (Gemperline, 2006). These algorithms have been used to identify unknown drugs (Farquharson et al., 2011), and in some cases determine relative concentrations in mixtures (Farquharson et al., 2017).

**Table 1:** Similar characteristic and unique SERS peaks for the 39 drugs measured using gold colloids and 785 nm excitation (see text for assignments).

**Drug, References**	**Similar characteristic peaks, cm**^****−1****^ **(see text for assignments)**												**Unique peaks**
**Spectral regions**	**500**	**600**	**700**	**800**	**900**	**1,000**	**1,100**	**1,200**	**1,300**	**1,400**	**1,600**	**1,700**	
Heroin (Gardner et al., 2013)	530	625						1,220		1,450	1,610		1,240
Morphine (Rana et al., 2010)	530	630						1,215		1,445	1,605		1,300
6-Acetylcodiene^*^	530	630						1,225		1,445	1,600		1,270
Codeine (Rana et al., 2010)								1,275		1,435	1,595		535
Hydrocodone (Rana et al., 2010)								1,275		1,435	1,605		510
Oxycodone^*^								1,275		1,435	1,595		975
Hydromorphone^*^ (Milne and Jhamandas, 1984)		625	755						1,320		1,615		835
Oxymorphone^*^ (Milne and Jhamandas, 1984)		630	750						1,320		1,610		1,685
Naltrexone^*^ (Milne and Jhamandas, 1984)		635	750						1,325		1,610		1,110
Naloxone^*^ (Milne and Jhamandas, 1984)		630		810				1,275					1,725
Buprenorphine		640		830						1,445	1,595		1,680
Norbuprenorphine^*^ (Milne and Jhamandas, 1984)		635		800							1,605		1,020
Methadone (Stanley, 2014)					915	1,000	1,190				1,595		675
Meperidine (Stanley, 2014)					910	1,000	1,195				1,600		585
Methylphenidate (Stanley, 2014)					910	1,000	1,185				1,600		495
Fentanyl (Hummel and Unterwald, 2002)						1,000, 1,030							465, 830
Carfentanil (Hummel and Unterwald, 2002)						1,000, 1,025							765
Remifentanil^*^						1,000, 1,025							600, 1,700
Cocaine (Carter et al., 2000)				890		1,000, 1,015							1,450
Benzoylecgonine^*^				885		995, 1,020							1,375
Cocaethylene^*^				890		1,000, 1,020							1,310
Amphetamine (Sägmüller et al., 2003)				815		1,000, 1,020		1,200					1,595
Methamphetamine (Sägmüller et al., 2003)				820		995, 1,020		1,200					1,570
Mephedrone^*^	^*^												805, 1,185
MDA (Derry et al., 2004)	530		720					1,250	1,370	1,435	1,620		1,195
MDMA (Derry et al., 2004)	530		720					1,250	1,365	1,435	1,615		1,025
MDME (Derry et al., 2004)	535		715					1,250	1,365	1,435	1,615		1,120
Diazepam (Cinta et al., 1999)					945	1,000	1,170				1,595		690
Temazepam (Cinta et al., 1999)					940	1,000	1,180				1,590		490
Oxazepam (Cinta et al., 1999)					935	1,000	1,175				1,600		1,475
Tetrahydrocannabinol (Sivashanmugan et al., 2019)							1,170						1,532
Cannabidiol^*^							1,170	1,290					1,345, 1,665
Cannabinol^*^								1,290					1,190
Nicotine (Pavel et al., 2003)	^*^												1,030, 1,660
Caffeine (Ricciotti and FitzGerald, 2011)					925			1,235	1,325		1,605	1,710	510, 1,290
Theophylline^*^					925			1,235	1,320		1,605	1,710	1,290
Acetaminophen (Vueba et al., 2008)									1,320				505, 1,535
Aspirin (Gemperline, 2006)									1,325				580, 1,400
Ibuprofen^*^ (Vueba et al., 2008)									1,325				1,660

* *Assignments based on similar drugs in this study*.

^**^The structure and spectra for this drug was different from any other.

## Conclusions

The SER spectra presented here are intended to aid researchers develop substrates, and companies develop products useful to first responders, police officers, medical point-of-care personnel, and military personnel. While a simple set of spectra have been presented, there are several important variables that can influence the actual measured spectra. We have attempted to eliminate two variables; laser wavelength and plasmonic metal type. However, sample concentration and pH can also significantly change the spectra. In most cases, the peaks will be at the same wavenumbers, but with different intensities. Buffers can be used to control the latter. The most challenging variable is the medium that the sample might be found. This includes numerous surface types, ranging from soil to clothing to illicit lab benches, numerous sample types, powders or liquids with other chemicals, and numerous body fluids from saliva to urine. Care must be taken in assessing the final analysis.

## Data Availability Statement

All datasets generated for this study are included in the manuscript/supplementary files.

## Author Contributions

SF directed the work and wrote this publication. CB designed the Raman analyzer used for these measurements. WS wrote the software to measure and analyse the spectra. CS prepared the gold nanoparticles and measured all of the drugs presented in this publication.

### Conflict of Interest

All authors were employed at Real-Time Analyzers, Inc. when this work was performed.
